# Immune response dynamics of SARS-CoV-2 vaccination in chronic lymphocytic leukemia individuals: a descriptive analysis

**DOI:** 10.3389/fimmu.2025.1571680

**Published:** 2025-06-06

**Authors:** Clara Sánchez-Menéndez, Alejandro Zurdo, Magdalena Corona, Elena Mateos de la Morenas, Sara Rodríguez-Mora, Guiomar Casado, Javier García-Pérez, Mayte Pérez-Olmeda, Susana Domínguez, María Aránzazu Murciano-Antón, Javier López-Jiménez, Valentín García-Gutiérrez, Mayte Coiras, Montserrat Torres

**Affiliations:** ^1^ Immunopathology and Viral Reservoir Unit, National Center of Microbiology, Instituto de Salud Carlos III, Madrid, Spain; ^2^ Hematology and Hemotherapy Service, Hospital Universitario Ramón y Cajal, Madrid, Spain; ^3^ PhD Program in Biomedical Sciences and Public Health, Universidad Nacional de Educación a Distancia (UNED), Madrid, Spain; ^4^ Biomedical Research Center Network in Infectious Diseases (CIBERINFEC), Madrid, Spain; ^5^ Faculty of Sciences, Universidad de Alcalá, Madrid, Spain; ^6^ AIDS Immunopathology Unit, National Center of Microbiology, Instituto de Salud Carlos III, Madrid, Spain; ^7^ Serology Laboratory, National Center of Microbiology, Instituto de Salud Carlos III, Madrid, Spain; ^8^ Family Medicine, Centro de Salud Arroyomolinos, Arroyomolinos, Madrid, Spain; ^9^ Family Medicine, Centro de Salud Doctor Pedro Laín Entralgo, Alcorcón, Madrid, Spain

**Keywords:** COVID-19 vaccine, cellular immune response, humoral immune response, chronic lymphocytic leukaemia, hematological malignancies

## Abstract

Chronic lymphocytic leukaemia (CLL) is a lymphoproliferative disorder of abnormal B-lymphocytes. Due to immune deregulation and therapy-related factors, CLL individuals face increased infection risks, making vaccination a priority. Although COVID-19 is no longer a global emergency, understanding vaccine responses in this vulnerable population, especially those undergoing active cancer treatments, remains critical for broader infectious disease prevention strategies. We have characterized the humoral and cellular immune response of SARS-CoV-2 vaccination elicited by CLL individuals under standard-of-care treatment and watch and wait (W&W) strategy compared with healthy subjects who received a three-dose regimen six months ago. Seroconversion rates varied between 81.8% and 71.4% in individuals under W&W and dropped to 28.6%-22.2% in those under treatment, with antibody titres and neutralizing activity following the same pattern, highlighting the impact of active therapies on vaccine immunogenicity. Analysis of B-cell dynamics revealed that individuals under W&W maintained the highest levels of total B cells (CD19+) throughout the study (up to 3.5-fold higher than healthy donors, p<0.0001). Basal naïve B cells were markedly reduced across CLL groups (up to 4.3-fold lower in treated *vs*. W&W, p<0.0001), while memory subsets expanded over time, particularly in the W&W cohort after booster vaccination. Additionally, we found that the actively treated CLL group exhibited higher levels of cytotoxic cells (including CD8+ T cells and NK cells) when compared to the W&W or the healthy population groups. However, none of these cell populations demonstrated an increased activation capacity. Moreover, the direct cytotoxic capacity of peripheral blood mononuclear cells (PBMCs) from CLL persons was also more efficient in the W&W group. Through our comprehensive characterization of both humoral and cellular immune responses in CLL individuals, this study provides insight into the complex immunological landscape following SARS-CoV-2 vaccination. Our detailed analysis supports the current vaccination strategy against SARS-CoV-2 for CLL patients, confirming its effectiveness and underscoring the importance of close monitoring and representing a significant advancement in our understanding of immune responses in hematological malignancies.

## Introduction

Chronic lymphocytic leukaemia (CLL) is a clonal lymphoproliferative disorder of abnormal B-lymphocytes in blood and lymphoid tissues. CLL is the main cause of leukaemia in the Western world and mainly affects the elderly, with a median age at diagnosis of 70 years and with multiple medical co-morbidities (1). The patients experience different clinical behaviors with diversity in disease course and outcome, ranging from an indolent disease which requires active surveillance or no treatment (“watch and wait” (W&W) strategy), to an aggressive disease characterized by progressive disease, resistance to therapy and poor overall survival (2). The immune deregulation inherent to CLL and patient- and therapy-related factors are the main reasons explaining the increased risk of infections in these patients, making prevention strategies such as vaccination a priority in this vulnerable population (3).

Coronavirus disease 2019 (COVID-19) caused by Severe Acute Respiratory Syndrome Coronavirus-2 (SARS-CoV-2) was declared a global public health threat by the World Health Organization on March 11, 2020 (4), but extensive research resulted in the most rapid and comprehensive vaccination program ever undertaken with more than 13 billion vaccine doses administered worldwide (5). Since the start of COVID-19 pandemic, CLL patients have been regarded as a vulnerable population due to the higher probability of progression to a severe infection, mainly those undergoing active cancer treatment (6, 7). Indeed, CLL patients present with higher rates of hospitalization, supplemental oxygen requirements, as well as higher intensive care unit (ICU) stays and mortality than cohorts without these lymphomas (8, 9). Key factors associated with this increased death risk are advanced age, poor performance status, low levels of platelets and elevated lactate dehydrogenase levels (8). In addition, SARS-CoV-2 infection can delay the chemotherapy treatment, leading to a poor outcome of CLL.

The European Medicines Agency (EMA) authorized six COVID-19 vaccines. mRNA-based Spikevax^®^ (Moderna) and Comirnaty^®^ (BioNTech-Pfizer) and vector-based Vaxzevria^®^ (Oxford/AstraZeneca) and Jcovden^®^ (Janssen) are approved for use in individuals with CLL (10). Vaccine efficacy studies describe lower seroconversion rates in CLL patients compared to healthy controls, and the lowest seropositivity and neutralization rates when compared to individuals with other oncohematological malignancies (11, 12). Moreover, those CLL individuals who are actively treated show poorer serological responses compared to those untreated (12–14). However, assessing humoral responses alone does not fully reflect the complexity of vaccine-induced immunity. T-cell-mediated immunity plays a crucial role in the control of viral infections, particularly in CLL individuals with impaired B-cell function. Several studies have shown that people with CLL, despite poor serological responses, can mount SARS-CoV-2-specific T cell responses with frequencies and functionality comparable to those of healthy donors that persist over time (14, 15). While most published research has focused on the humoral response, there is comparatively less evidence regarding the cellular immune response, despite its relevance in antiviral protection. Given the immune dysregulation in CLL, evaluating both humoral and cellular responses is essential to understanding the vaccine-induced protection in this population.

In this study, we characterized humoral and cellular immunity elicited by SARS-CoV-2 vaccination in people with CLL under standard-of-care treatment and watch or wait (W&W) strategy who received a three-dose schedule six months ago compared with healthy donors. The information provided in this study may be valuable to guide clinical decision-making in the management of CLL individuals.

## Materials and methods

### Study population

A prospective, observational, longitudinal study with 25 individuals with CLL recruited at the Hematology and Hemotherapy Service of Hospital Universitario Ramón y Cajal (Madrid; Spain) between March 2021 and February 2022, under standard-of-care treatment and W&W strategy. Sample size was calculated based on expected differences in both humoral and cellular immune responses after vaccination. Previous studies reported seroconversion rates of 90% in healthy controls, 70% in CLL patients under W&W, and 40% in those receiving treatment. To detect a minimum difference of 50 percentage points between groups, with 80% power and a two-sided α of 0.05, a minimum of 12 participants *per* group was required. For cellular responses, previous studies showed responses in 80-90% of healthy controls and 50% of treated CLL patients; thus a sample size of 10 participants *per* group was considered sufficient under similar assumptions. Participants were 18 years or older without previous SARS-CoV-2 infection and candidates for a three-dose, full vaccination schedule. Blood samples and clinical data were collected as follows: before COVID-19 vaccination (Basal sample), one month after receiving the second vaccine dose (Sample 1), one month after receiving the booster (Sample 2), and six months after having received the booster (Sample 3).

Twelve healthy donors were recruited from the Primary Healthcare Center Doctor Pedro Lain Entralgo (Madrid, Spain) with no previous history of SARS-CoV-2 infection and candidates to receive the same three doses, full vaccination schedule against SARS-CoV-2. Samples collection schemed similarly to CLL participants.

### Ethical statement

All individuals gave informed written consent to participate in the study. Confidentiality and anonymity were protected by current Spanish and European Data Protection Acts. Protocol for this study was performed under the Helsinki Declaration and it was approved by the Ethics Committees of Instituto de Salud Carlos III (protocol CEI PI 32_2020-v2) and the participating centers (protocols 122–20 and 20/20).

### Samples processing and materials

Peripheral blood samples were collected in EDTA Vacutainer tubes (Becton Dickinson, Madrid, Spain) and processed by Ficoll-Hypaque (Pharmacia Corporation, North Peapack, NJ) density gradient centrifugation to isolate peripheral blood mononuclear cells (PBMCs) and plasma, which were cryopreserved until analysis. Raji (ATCC CCL-86) and HEK-293T (ATCC CRL-3216) cell lines were provided by the existing collection of Instituto de Salud Carlos III (Madrid, Spain) and Vero E6 (African green monkey kidney) cell line (ECACC 85020206) was kindly provided by Dr. Antonio Alcami (CBM Severo Ochoa, Madrid). Vero E6 and HEK-293T cells were cultured in DMEM supplemented with 10% FCS, 2 mM L-glutamine, and 100 units/ml penicillin/streptomycin (Lonza, Basel, Switzerland). Raji cells were cultured in RPMI-1640 medium with the same supplements.

### SARS-CoV-2 serology

Euroimmun Anti-SARS-CoV-2 ELISA Assay (Euroimmun, Germany) was employed to identify IgG antibodies against SARS-CoV-2 spike (S) protein in plasma according to the manufacturer’s instructions.

### Pseudovirus neutralization assays

A SARS-CoV-2 neutralization assay was used to identify the presence of neutralizing antibodies as previously described (16–19). Briefly, 4-fold serial dilutions (1/32 to 1/8192) of heat inactivate (1 hour at 56°C) IgG-positive samples were added to Vero E6 cells infected with equal amounts of one-cycle pseudoviruses D614 and G614 (100ng p24 Gag/well). The titres of neutralizing antibodies were calculated as 50% inhibitory dose (ID50) as the highest plasma dilution that resulted in a 50% reduction of luciferase activity compared to a control.

### Direct cell-mediated cytotoxicity assay against pseudotyped SARS-CoV-2-infected cells

Direct cell-mediated cytotoxicity (DCC) against SARS-CoV-2-infected cells of PBMCs was performed using one-cycle pseudoviruses D614 and G614 infecting Vero E6 cells. After 48 hours, cells were co-cultured with participants’ PBMCs (1:1 ratio). Vero E6 monolayer was dissociated with trypsin-EDTA solution (Sigma Aldrich-Merck, Germany), and caspase-3 activity was quantified by luminescence using Caspase-Glo 3/7 Analysis Kit (Promega). Then, cells were lysed, and viral infectivity was assessed by measuring *Renilla* luciferase activity as described above. PBMCs were collected from the Vero E6 supernatants and cytotoxic cell populations such as Natural Killer (NK), NKT-like and Tγδ+ cells were analyzed employing the conjugated antibodies: CD3-PE, CD56-BV605, CD16-PercP, CD8-APC H7, CD107a-PE-Cy7, and TCRγδ-FITC (BD Biosciences; San Jose, CA). Data was acquired on a BD LSRFortessa X-20 (BD Biosciences, USA) and analyzed with Flow Jo software v10.9.0 (Tree Star Inc., USA).

### Antibody-dependent cellular cytotoxicity assay

Antibody-dependent cytotoxic activity (ADCC) of participants’ PBMCs was measured using the NK-sensitive target Raji cell line. Cells were first labelled with PKH67 Green Fluorescent Cell Linker (Merck KGaA, Germany), coated with rituximab (50µg/ml) (Selleckhem, Houston, TX), and co-cultured with PBMCs (1:1 ratio). Raji cell apoptosis was determined by staining with Annexin V conjugated with phycoerythrin (PE) (Immunostep, Spain). Cytotoxic cell populations in the co-culture supernatants were analyzed by flow cytometric as previously described.

### Characterization of B lymphocyte phenotypes

Subpopulations of B cells (CD3-CD19+) were characterized by flow cytometry after staining with antibodies CD3-PE, CD10-BV421, CD19-BV711, CD20-AlexaFluor700, CD21-FITC, CD27-PercP-Cy5.5 (BD Biosciences, CA) to identify: immature or transitional cells (CD10+ CD27-); naïve B cells (CD10-CD27-CD21^high^); tissue-like memory cells (CD10-CD27-CD21^low^); resting memory cells (CD10-CD27+CD21^high^); activated memory cells (CD10-CD27+CD21^low^); and plasmablasts (CD27++CD20-CD21^low^) (20). As previously described, data acquisition was performed in a BD LSRFortessa X-20 flow cytometer and FlowJo was used for data analysis.

### Statistical analysis

Statistical analysis was performed using GraphPad Prism v10.2.3 (GraphPad Software Inc., San Diego, CA). Quantitative variables were expressed as median ± 25th and 75th percentiles (Q1, Q3), and categorical variables as percentages. Comparisons were performed employing chi-square, Student t-test, Wilcoxon matched-pairs signed rank, or Mann-Whitney tests as appropriate. P values (p) < 0.05 were considered statistically significant in all comparisons.

## Results

### Study individuals’ characteristics

Demographical and clinical descriptions of the participants are shown in **Table 1**. The median age of the healthy donors was 71 years old (IQR, 49-71.0) and five (41.7%) were male. The median age of participants in the W&W cohort was 77 years old (IQR: 66.5-81) and 74 years old (IQR: 66.0-80.5) in the treated cohort. In both CLL groups, the majority (63.6% and 71.4%, respectively) of participants were male. Comirnaty^®^ (Pfizer-BioNTech) was the vaccine most frequently administrated by both healthy donors (91.7%) and CLL individuals (45.45% and 65.2%). None of the participants reported side effects after COVID-19 vaccination. Three (25%) participants from the healthy donors group reported breakthrough infections *versus* five (45.5%) participants from the W&W CLL cohort and nine (64.3%) from the treated CLL cohort (p=0.13). Two (16.7%) participants of treated CLL group developed severe COVID-19 with pulmonary damage characterized by bilateral infiltrates, as well as dyspnea and hypoxia that require hospitalization and supplemental oxygen (21). Regarding comorbidities, hypertension (41.7%; p=0.045) and dyslipidemia (33.3%; p=0.055) were most prevalent in the healthy donor group, compared to three subjects (21.4%) and one subject (7.1%), respectively, in the treated CLL group. No cases of either condition were found in the Watch & Wait (W&W) group. Among CLL participants the time since diagnosis was 8.5 years (IQR: 4.3-9.8) for the W&W cohort and 10 years (IQR: 6-15) for the treated cohort, and the median age at diagnosis was 64 years (IQR: 60.0-72.5; IQR: 59.0-73.0) in both groups. Bruton tyrosine kinase inhibitors (BTKi) were the most commonly used therapeutic intervention, administered in 64.3% of cases. The treated CLL cohort demonstrated a significantly higher rate of secondary cancers compared to the W&W cohort (42.8% *vs* 18.2%, p=0.024). Additionally, unmutated IGHV (immunoglobulin heavy chain variable) status was exclusively found in the treated group (57.1% *vs* 0%; p=0.003).

**Table 1:** Demographics and clinical characteristics of participants in the study.

Characteristics	Healthy donors	W&W CLL cohort	Treated CLL cohort	p-value
	n = 12	n=11	n=14	
Age, median (IQR)— yr	71 (49-71)	77 (66.5-81)	74.5 (66-80.5)	0.003
Gender, — no. (%)				
Male	5 (41.7)	7 (63.6)	10 (71.4)	0.34
Timing of samples, median (IQR)—days				
Second dose to the second sample	23 (23-27)	35 (32-38)	31 (28-34)	0.001
Booster dose to the third sample	30 (27-34)	32 (28-38)	37 (32-47)	0.20
Booster dose to the fourth sample	143 (135-152)	193 (186-260)	202 (195-327)	0.0003
COVID-19 vaccines— no. (%)				
Spikevax (Moderna)	–	4 (36.4)	3 (21.4)	0.07
Comirnaty (Pfizer-BioNTech)	11 (91.7)	5 (45.45)	9 (64.2)	0.06
Vaxzevria (AstraZeneca)	–	2 (18.2)	2 (14.3)	0.43
Jcovden (Janssen)	1 (8.3)	–	–	1
Breakthrough infections— no. (%)				
Yes	3 (25)	5 (45.45)	9 (64.2)	0.13
Coexisting conditions— no. (%)				
Hypertension	5 (41.7)	–	3 (21.4)	0.045
Dyslipidemia	4 (33.3)	–	1 (7.1)	0.055
Diabetes	1 (8.3)	–	2 (14.3)	1
Obesity	3 (25)	–	1 (7.1)	0.18
Atrial Fibrillation	2 (16.7)	1 (9.1)	1 (7.1)	0.82
Obstructive Sleep Apnea	2 (16.7)	–	1 (7.1)	0.49
Time from CLL diagnosis, median (IQR)—yr	N/A	8.5 (4.3-9.8)	10 (6-15)	0.47
Age at diagnosis CLL, median (IQR)— yr	N/A	64.0 (60.0-72.5)	64.0 (59.0-73.0)	0.81
Immunomodulatory treatment— no. (%)				
Anti-CD20 therapy	N/A	–	1 (7.1)	1
BTKi	N/A	–	9 (64.3)	0.001
BCL2i	N/A	–	3 (21.4)	0.23
BTKi/BCL2i	N/A	–	1 (7.1)	1
Previous CLL treatments— no. (%)	N/A	1 (9)	7 (50)	0.042
IGHV status— no. (%)				
Mutated	N/A	5 (45.45)	3 (21.4)	0.39
Unmutated	N/A	–	8 (57.1)	0.003
Unknown	N/A	6 (54.5)	3 (21.4)	0.11
Coexisting conditions— no. (%)				
Secondary cancers	N/A	2 (18.2)	6 (42.8)	0.024

BCL2i, B-cell lymphoma 2 inhibitors; BTKi, Bruton tyrosine kinase inhibitors; CD20, cluster of differentiation 20; IGHV, immunoglobulin heavy chain variable; IQR, interquartile range; N/A, not applicable; no., number; OSA, Obstructive Sleep Apnea; W&W, watch and wait.

### Blood sample collection

The time from the vaccination and the collection of the samples among the participants is shown in **Supplementary Figure 1**. Among the healthy donor group, Sample 1 was collected with a median of 23 days (IQR: 23-27) after the second dose, Sample 2 a median of 30 days (IQR: 27-34) after the booster, and Sample 3 a median of 143 (IQR: 135-152) after the booster. In CLL patients, Sample 1 was collected with a median of 33 days (IQR: 30-38) and Sample 2 and 3, a median of 34 (IQR: 29-43) and 202 days (IQR: 190-319), respectively (**Table 1**).

### Participants of the treated CLL cohort presented the lowest seroconversion rate after vaccination

**Figure 1:**
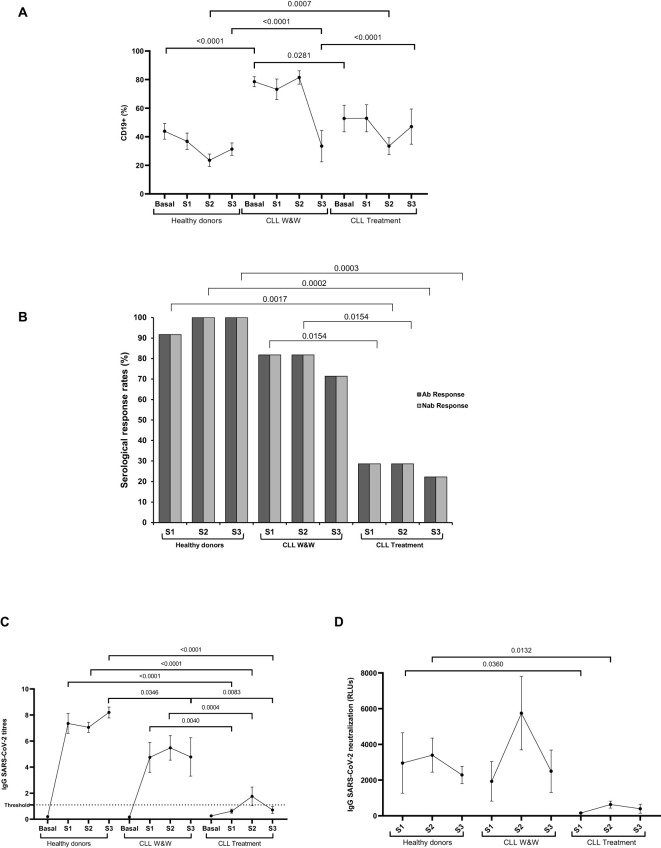
Serological response against COVID-19 vaccine in plasma of healthy donors and chronic lymphocytic leukemia patients during the study. **(A)** Total B cell levels in PBMCs of healthy donors and chronic lymphocytic leukaemia patients on treatment and under the watch and wait (W&W) strategy. **(B)** IgG and neutralizing antibody positivity rates. **(C)** IgG titres in plasma from the healthy donors and chronic lymphocytic leukaemia participants on treatment and under the watch and wait (W&W) strategy. **(D)** Neutralizing antibody titres at 50% inhibition (NT50) against SARS-CoV-2 of plasma from the healthy donors and chronic lymphocytic leukaemia individuals on treatment and under the W&W strategy. Each dot in the graphs corresponds to the mean and the vertical lines correspond to the standard error of the mean (SEM). Statistical significance was calculated using Fisher exact test, Wilcoxon signed-rank test, Mann-Whitney U and Student t-test, as appropriate. Ab, Antibody; Nab, Neutralizing antibody.

The highest levels of B cells (CD19+) were observed in W&W cohort in Basal, Sample 1 and Sample 2 (1.8-fold; p<0.0001; 2.0-fold; p=0.0007; 3.5-fold; p<0.0001, respectively) compared to healthy donors, and in Basal and Sample 2 (1.5-fold; p=0.0281;1.4-fold; p<0.0001) compared to treated participants (**Figure 1A**).

Most (91.7%) healthy donors showed detectable levels of IgGs against S protein in Sample 1 and all (100%) participants in Samples 2 and 3. In CLL cohorts, seroconversion rates ranged from 81.8% in Samples 1 and 2 to 71.4% in Sample 3 among individuals in the W&W cohort and from 28.6% in Samples 1 and 2 to 22.2% in Sample 3 in the treated cohort (**Figure 1B**). Titres in Samples 1, 2 and 3 from W&W group were 7.7-(p=0.0004), 3.1-(p=0.004) and 6.7-fold (p=0.0083) lower compared with healthy donors. Similarly, the titres in the treated group were significantly reduced 11.2-, 3.6- and 11.3-fold (p<0.0001) when compared to those reported in healthy donors (**Figure 1C**).

**Figure 2:**
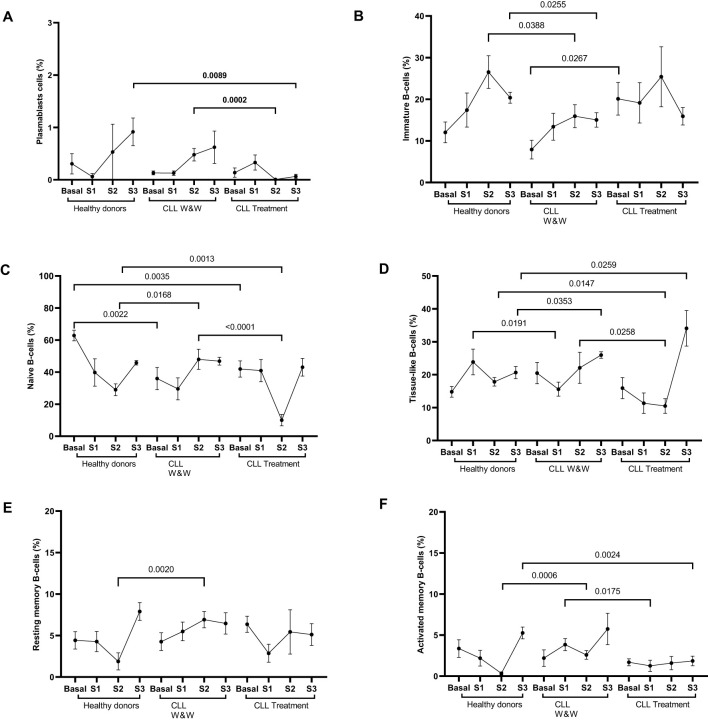
Analysis of the distribution of lymphocyte B cell populations from healthy donors and chronic lymphocytic leukaemia patients during the study. **(A)** Levels of plasmablasts (CD27++CD20-CD21low) in PBMCs from individuals of the cohorts. **(B)** Levels of immature (CD10+CD27-) cells in PBMCs from individuals of the groups. **(C)** Levels of basal naïve B cells (CD10-CD27-CD21high) in PBMCs from individuals of the cohorts. **(D)** Levels of tissue-like memory cells (CD10-CD27-CD21low) in PBMCs from individuals of the groups. **(E)** Levels of resting memory cells (CD10-CD27+CD21 high) in PBMCs from individuals of the groups. **(F)** Levels of activated cells (CD10-CD27+CD21low) in PBMCs from individuals of the cohorts. Each dot in the graphs corresponds to the mean and the vertical lines correspond to the standard error of the mean (SEM). Statistical significance was calculated using Wilcoxon signed-rank test, Mann-Whitney U and Student t-test, as appropriate.

Those participants with detectable levels of IgGs were further analyzed to evaluate the neutralization response and in all (100%) cases developed neutralizing antibodies (**Figure 2B**). The lowest neutralizing capacity against SARS-CoV-2 was shown in Samples 1 and 2 of the treated cohort, being 18.9-(p=0.036) and 5.4-fold (p=0.0132) lower than showed in healthy donors, respectively. Interestingly, participants under the W&W strategy showed neutralizing capacities similar to those reported in the healthy donors throughout the study (**Figure 1D**).

### Alterations in the composition of B-cell subpopulations in CLL cohorts

The analysis of B-cell subpopulations found an increase in the levels of plasmablasts (CD27++CD20-CD21low) in healthy donors and W&W participants throughout the study, in contrast to treated CLL participants which presented only a slight increase in Sample 1 (**Figure 2A**). The subpopulation of immature (CD10+CD27-) cells showed similar levels in healthy donors and treated participants, while the levels in W&W participants decreased in Sample 2 (1.7-fold; p=0.0388) and Sample 3 (1.4 fold; p=0.0255) when were compared to healthy donors (**Figure 2B**). Basal naïve B cells (CD10-CD27-CD21high) were significantly (p=0.0035 and p=0.0022) lower in CLL participants compared to those reported in the healthy donor group. Sample 2 levels were significantly (2.9-fold; p=0.0013 and 4.3-fold; p<0.0001) reduced in treated participants compared to healthy and W&W participants, respectively (**Figure 2C**). Tissue-like memory cells (CD10-CD27-CD21low) levels of W&W participants in Sample 1 were significantly lower than those observed in healthy participants (2.3-fold; p=0.0191), although increased significantly (1.7-fold; p=0.0353) in Sample 3. Similarly, treated CLL participants showed reduced levels (1.7-fold; p=0.0147) compared to healthy donors in Sample 2, although increased (1.7-fold; p=0.0259) at the end of the study (**Figure 2D**). Regarding memory cells, both resting (CD10-CD27+CD21high) and activated cells (CD10-CD27+CD21low) showed increased levels (p=0.002 and p=0.0006, respectively) in participants in the W&W group when compared to the healthy donor group one month after the booster dose (Sample 2) (**Figures 2E, F**). Activated memory cells were also significantly (p=0.0175) increased in W&W CLL individuals in Sample 1 compared to treated CLL participants.

### Similar levels of antibody-dependent cellular cytotoxic response were observed between cohorts

**Figure 3:**
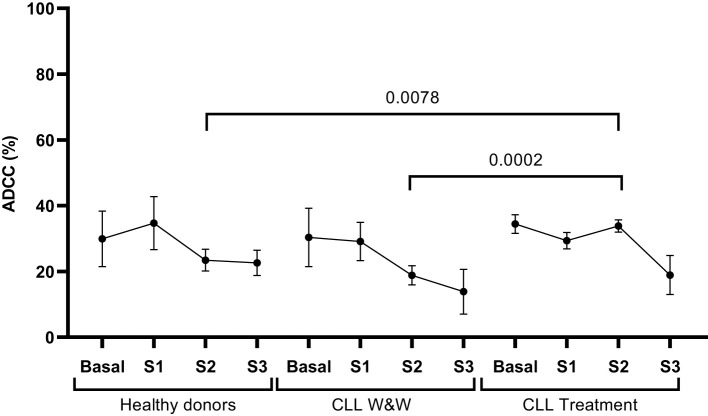
Analysis of ADCC response of PBMCs from healthy donors and chronic lymphocytic leukaemia individuals during the study. Quantification of the expression of phosphatidylserine in the surface of rituximab-coated Raji cells co-cultured with PBMCs isolated from healthy donors and chronic lymphocytic leukaemia individuals on treatment and under the watch and wait (W&W) strategy after staining with Annexin V. Each dot in the graph corresponds to mean and the vertical lines correspond to the standard error of the mean (SEM). Statistical significance was calculated using Wilcoxon signed-rank test, Mann-Whitney U and Student t-test, as appropriate.

PBMCs from all participants showed antibody-mediated cellular cytotoxicity (ADCC) activity against rituximab-coated Raji cells as target. We did not observe differences in the levels between the groups, except for treated participants whose levels were significantly (1.8-fold; p=0.0002; 1.6-fold; p=0.0078, respectively) increased compared with W&W participants and healthy donors in Sample 2 (**Figure 3**).

### Lower direct cellular cytotoxicity from treated CLL cohort

**Figure 4:**
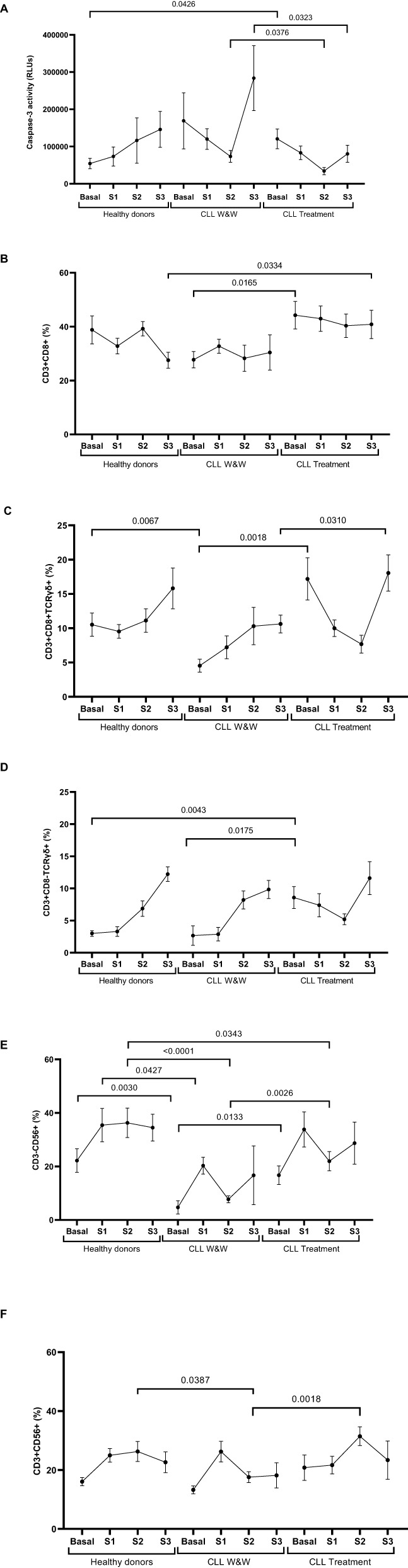
Analysis of DCC response of PBMCs from healthy donors and chronic lymphocytic leukaemia individuals during the study. **(A)** DCC was assessed by measuring the activity of caspase-3 in pseudotyped-SARS-CoV-2-infected Vero E6 cells co-cultured with PBMCs from healthy donors and chronic lymphocytic leukaemia individuals on treatment and under the watch and wait (W&W) strategy. **(B)** Total levels of CD3+CD8+ cells in PBMCs from individuals of the cohorts. **(C)** Total levels of CD3+CD8+TCRgd+ cells in PBMCs from individuals of the cohorts. **(D)** Total levels of CD3+CD8-TCRgd+ cells in PBMCs from individuals of the cohorts. **(E)** Total levels of CD3-CD56+ cells (NK) in PBMCs from individuals of the cohorts. **(F)** Total levels of CD3+CD56+ cells (NKT-like) in PBMCs from individuals of the cohorts. Each dot in the graphs corresponds to the mean and the vertical lines correspond to the standard error of the mean (SEM). Statistical significance was calculated using Wilcoxon signed-rank test, Mann-Whitney U and Student t test, as appropriate.

The specific DCC activity of PBMCs from the treated participants was reduced 2.1-fold (p=0.0376) and 3.5-fold (p=0.0323) in comparison with W&W participants one and six months after the booster dose (Samples 2 and 3), respectively (**Figure 4A**). No significant differences were observed in the total levels of CD8+ T cells, except for higher levels in Basal sample among treated CLL participants compared to those in the W&W strategy (1.6-fold; p=0.0165), and higher levels in Sample 3 in the treated CLL cohort compared to healthy donors (1.5-fold; p=0.0334) (**Figure 4B**). The study of the highly cytotoxic CD3+CD8+TCRγδ+ population showed basal levels 3.8-fold lower (p=0.0018) in the W&W participants *versus* treated participants, and levels 2.3-fold lower (p=0.0067) compared to the healthy donors. The levels remained significantly (-1.7-fold; p=0.0310) reduced in Sample 3 compared to treated participants (**Figure 4C**). Similarly, we found differences in the CD3+CD8-TCRγδ+ population, with levels significantly higher in Basal sample in treated CLL participants compared to those in W&W (3.2-fold; p=0.0043) and healthy donor groups (2.9-fold; p=0.0175) (**Figure 4D**). The activation of these cytotoxic cells, assessed through the expression of the surface degranulation marker CD107a, showed no significant differences among the cohorts (**Supplementary Figure 2**).

The analysis of NK cells (CD3-CD56+) revealed lower levels in the W&W cohort compared to the healthy donors, with levels 4.7-fold lower (p=0.0030) in Basal sample, 2.0-fold lower (p=0.0427) in Sample 1 and 4.7-fold lower (p<0.0001) in Sample 2 (**Figure 4E**). Similarly, NK cell levels were also decreased in W&W cohort compared to treated cohort in Basal sample (3.5-fold; p=0.0133) and Sample 2 (2.8-fold; p=0.0026). NKT-like cell (CD3+CD56+) levels were significantly lower in W&W participants *versus* treated participants (1.8-fold; p=0.018) and healthy donors (1.2-fold; p=0.0387) in Sample 2 (**Figure 4F**). No significant differences in CD107a expression were found between cohorts (**Supplementary Figure 3**).

## Discussion

Chronic lymphocytic leukaemia (CLL) is characterized by an immunodeficiency that increases the risk of developing severe infections, especially those of viral origin. Therefore, the increased risk of morbidity and mortality from the infections could benefit from vaccination. As a result, International Medicines Agencies approve and recommend antibacterial and antiviral prophylactic vaccines for CLL patients (10, 22). However, the mechanisms underlying the immunodeficiency related to their primary disease and those caused by the treatments may impair the immune response to vaccines, making it essential to monitor the efficacy of the vaccination.

According to other publications, our work identified lower seroconversion rates in CLL patients than in healthy subjects, reflecting the malignancy of the CD5+ B cells that causes deficient humoral immune response in this population (9, 23, 24). However, seropositive rates in individuals of our W&W cohort (71.4%-81.8%) were similar to those reported in previous studies (14, 25, 26) confirming lower vaccination responses than those achieved by individuals with other oncohematological malignancies, likely due to B-lymphocyte dysfunction in CLL (8, 24). The lowest seroconversion rates (22.2%-28.6%) were observed in those patients undergoing active treatment, with the majority (61.3%) receiving BTKi. Interestingly, in our cohort, those participants treated with BCL2i achieved seroconversion rates higher than the participants treated with BTKi. The values are consistent with previous studies (25, 27), and they further confirm that active therapy is an independent factor associated with a poor immune response (25–27). Furthermore, our findings align with certain studies that point out that treatment with BTKi further compromises the humoral response to the vaccine compared to other regimens (9, 28). The mechanism of the BTKis to alter the humoral response is not clear, although may be due to the disruption in signaling pathways in normal B-cell, and/or off-target effects on other kinases affecting CD4+ T cell function (8). The impaired humoral response in treated CLL participants was also reflected in the antibody titres, which failed to reach the positive threshold *versus* the participants of the W&W cohort, whose titres were comparable to those reported in the healthy donors. Along with active treatment, the disruptions in the maturation and functionality of B cells associated with the disease may lead to reduced and less effective antibody titres (25, 27). Nevertheless, the seropositivity and antibody titres after the booster decreased much more rapidly in CLL participants compared to the general population. Among others, an impaired rate of antibody turnover or difficulties in generating memory B cells consequence of the diminished humoral response could be related to maintaining a long-lasting antibody response. As expected, neutralization rates and titres were lower in treated CLL participants in comparison with W&W CLL participants, whose values were comparable to those detected in healthy donors, suggesting a good stimulation of helper T cells and the development of immune memory, which enhances the protection in this CLL cohort.

On the other hand, and as expected, participants in the W&W cohort exhibited significantly higher levels of B cells (CD19+) compared to both treated CLL participants and healthy donors. The levels of plasmablasts of the participants in the W&W cohort were similar to those of healthy donors but were markedly reduced in the participants in the treated cohort. This reduction also explained the significantly diminished capacity for total IgG production and neutralizing ability in these patients compared to those in the W&W strategy. The assessment of T-cell immunity in response to vaccination was analyzed by evaluating the direct cytotoxic capacity of PBMCs, revealing a superior cytotoxic capacity in the individuals of the W&W cohort compared to those under treatment. This finding is particularly noteworthy given that the treated CLL cohort exhibited higher levels of cytotoxic cells when compared to both W&W and control groups. Specifically, we observed elevated populations of CD8+ T cells, CD8+ Tγδ lymphocytes, NK cells, and NKT cells. However, despite their increased numbers, none of these cellular populations demonstrated enhanced activation capacity, as measured by the expression of the degranulation marker CD107a. Previous studies reported that CLL individuals develop T-cell immunity vaccination, despite anti-B-cell treatment that is mainly associated with elevated CD8+ levels, which have been related to improved COVID-19 outcomes (29–31). Interestingly, despite the reduced DCC activity in treated CLL cohort, ADCC responses remained preserved. Given the potential compensatory role of Fc-mediated functions in antiviral defense, particularly in immunocompromised individuals (32), this preserved activity might contribute to a protective effect of vaccination in this cohort. Despite observing a decline in the cytotoxic activity after the second dose and the booster, a subsequent increase in the cellular response six months after the booster suggests a potential delay in the differentiation phase of memory response in CLL individuals and/or the occurrence of breakthrough infections. Although these infections were mild in our cohorts, they might have contributed to inducing a stronger cellular response in the absence of a fourth dose. Our CLL participants showed a higher breakthrough infection rate (53%) compared to previous studies (15-21%) (33), even though mild and resolved without severe complications.

Over time, we observed a progressive decline in humoral responses in both CLL cohorts, reflected in both antibody titres and neutralization rates, particularly in the treated cohort. However, the cellular response in the W&W participants remained detectable with signs of recovery at six months, suggesting a durable cellular memory. This is in line with previous findings in which diminished humoral response does not necessarily compromise protection against severe disease, as T-cells can play a crucial role in long-term defense in immunocompromised individuals (27, 34). Furthermore, although breakthrough infections were more frequent in the treated cohort than healthy controls, these were mostly mild, possibly reflecting partial protection mediated by cellular responses.

Our results align with studies in immunocompromised populations, individuals with cancer under active therapy, and individuals with inborn errors of immunity (35–37). These populations develop detectable antibody responses, although those receiving B-cell-depleting agents or intensive chemotherapy exhibit a markedly diminished humoral response, similar to our BTKi-treated cohort. Moreover, detectable or relatively preserved T-cell responses observed in these populations, even in the presence of limited humoral immunity, may still contribute to an adequate vaccine response and/or protection against severe disease (35)

A potential limitation of the study is related to clinical parameters that may affect the participants’ outcomes, such as the IGHV status and secondary cancers in CLL participants, or comorbidities such as hypertension in healthy donors that have not been considered. However, given the homogeneous nature of our population, is unlikely these factors have substantially impacted.

The impaired immune responses observed in our CLL cohorts following vaccination likely stem from multiple interrelated mechanisms characteristic of this disease. At the cellular level, the malignant B-cell clone not only competes with normal B cells for survival factors but also occupies lymphoid niches, disrupting germinal center formation and impairing antibody maturation (38), factors explaining the lower antibody levels we detected compared to healthy subjects, even after a booster dose. The immunosuppressive microenvironment created by CLL cells secreting cytokines such as IL-10 and TGF-β further dampens both B- and T-cell function (39), potentially contributing to the gradual decline in antibody and T-cell immunity we observed over six months. Treatment significantly exacerbated these defects, as demonstrated by the weaker immune response in treated CLL participants compared to W&W participants: BTK inhibitors block critical B-cell receptor signaling needed for antibody generation, anti-CD20 antibodies deplete the very B cells required for humoral immunity, and BCL-2 inhibition can alter the metabolic fitness of both humoral and cellular immune compartments (40, 41). Despite these profound immunological challenges, our findings reveal that a two-dose full vaccination regimen followed by a booster dose still elicits protective immune responses in CLL individuals that remain detectable six months post-vaccination, particularly in W&W individuals, albeit at lower levels than in healthy subjects, highlighting how this dysregulated immune landscape affects vaccine efficacy while still allowing for meaningful protection. Beyond confirming previously described impaired humoral and cellular responses after vaccination in CLL individuals, our study provides novel evidence by characterizing for the first time the distribution of B-cell subpopulations and the functional assessment of cytotoxic immune responses, both antibody-dependent cellular cytotoxicity (ADCC) and direct cellular cytotoxicity (DCC) and supported the validity of the current vaccination strategy for CLL individuals, confirming its effectiveness and underscoring the importance of close monitoring, although we could not rule out that higher susceptibility to breakthrough infections in these individuals was not acting as an additional booster. Our comprehensive analysis of CLL individuals reveals critical insights into immune function under different treatment conditions, and our findings not only validate current SARS-CoV-2 vaccination strategies for CLL patients but also provide valuable insights for future vaccination approaches across various infectious diseases.

## Data Availability

The raw data supporting the conclusions of this article will be made available by the authors, without undue reservation.
