# Correction: Immune response dynamics of SARS-CoV-2 vaccination in chronic lymphocytic leukemia individuals: a descriptive analysis

**DOI:** 10.3389/fimmu.2025.1706723

**Published:** 2025-09-19

**Authors:** Clara Sánchez-Menéndez, Alejandro Zurdo, Magdalena Corona, Elena Mateos de la Morenas, Sara Rodríguez-Mora, Guiomar Casado, Javier García-Pérez, Mayte Pérez-Olmeda, Susana Domínguez, María Aránzazu Murciano-Antón, Javier López-Jiménez, Valentín García-Gutiérrez, Mayte Coiras, Montserrat Torres

**Affiliations:** ^1^ Immunopathology and Viral Reservoir Unit, National Center of Microbiology, Instituto de Salud Carlos III, Madrid, Spain; ^2^ Hematology and Hemotherapy Service, Hospital Universitario Ramón y Cajal, Madrid, Spain; ^3^ PhD Program in Biomedical Sciences and Public Health, Universidad Nacional de Educación a Distancia (UNED), Madrid, Spain; ^4^ Biomedical Research Center Network in Infectious Diseases (CIBERINFEC), Madrid, Spain; ^5^ Faculty of Sciences, Universidad de Alcalá, Madrid, Spain; ^6^ AIDS Immunopathology Unit, National Center of Microbiology, Instituto de Salud Carlos III, Madrid, Spain; ^7^ Serology Laboratory, National Center of Microbiology, Instituto de Salud Carlos III, Madrid, Spain; ^8^ Family Medicine, Centro de Salud Arroyomolinos, Arroyomolinos, Madrid, Spain; ^9^ Family Medicine, Centro de Salud Doctor Pedro Laín Entralgo, Alcorcón, Madrid, Spain

**Keywords:** COVID-19 vaccine, cellular immune response, humoral immune response, chronic lymphocytic leukaemia, hematological malignancies

An incorrect grant number was provided for Instituto de salud Carlos III. The correct number is “PI24/00861” instead of “PI24/00864”.

The original version of this article has been updated.

