# Supplementary material for: Immune response dynamics of SARS-CoV-2 vaccination in chronic lymphocytic leukemia individuals: a descriptive analysis
Source: Front Immunol. 2025 Jun 6;16:1571680. doi: 10.3389/fimmu.2025.1571680 (PMC12179140; doi:10.3389/fimmu.2025.1571680)
Supplement: Supplementary file 1 [file Image1.pdf]

Supplemental Figure 1. Vaccination status is shown along with the sample number. A baseline sample was collected before the first dose of the vaccine. Sample 1 collected a median from 23 days and up to 35 days after the second dose, Sample 2 collected a median from 30 days and up to 37 days after the booster dose, and Sample 3 collected a median from 143 days and up to 202 days after the booster.

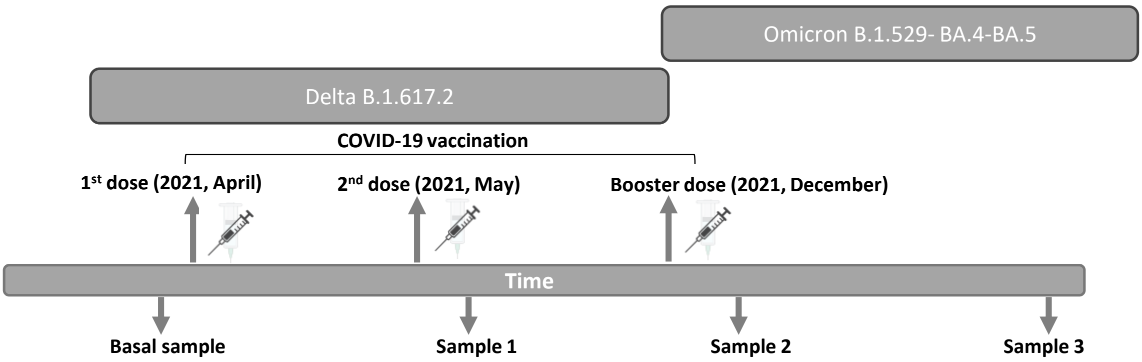

Group of study	Timing of blood samples, median (IQR)—days		
	Sample 1	Sample 2	Sample 3
W&W CLL cohort (n=11)	35 (32-38)	32 (28-38)	193 (186-260)
Treated CLL cohort (n=14)	28 (31-34)	37 (32-47)	202 (195-327)
Healthy donors (n=12)	23 (23-27)	30 (27-34)	143 (135-152)

CLL: Chronic lymphocytic leukaemia; W&W: Watch and wait strategy
