# Supplementary material for: Immune response dynamics of SARS-CoV-2 vaccination in chronic lymphocytic leukemia individuals: a descriptive analysis
Source: Front Immunol. 2025 Jun 6;16:1571680. doi: 10.3389/fimmu.2025.1571680 (PMC12179140; doi:10.3389/fimmu.2025.1571680)

**Supplemental Figure 2.** Expression levels of the degranulation marker CD107a in cytotoxic cell populations of PBMCs from healthy donors and chronic lymphocytic leukaemia patients on treatment and under the watch and wait (W&W) strategy. The expression of CD107a was analyzed by flow cytometry in the surface of CD3+CD8+ cells (A), CD3+CD8+TCRgd+ cells (B), CD8-TCRgd+ cells (C) of PBMCs isolated from individuals of the three groups. Each dot in the graphs corresponds to mean ± SEM. Statistical significance was calculated using Wilcoxon signed-rank test, Mann-Whitney U and Student t test, as appropriate.

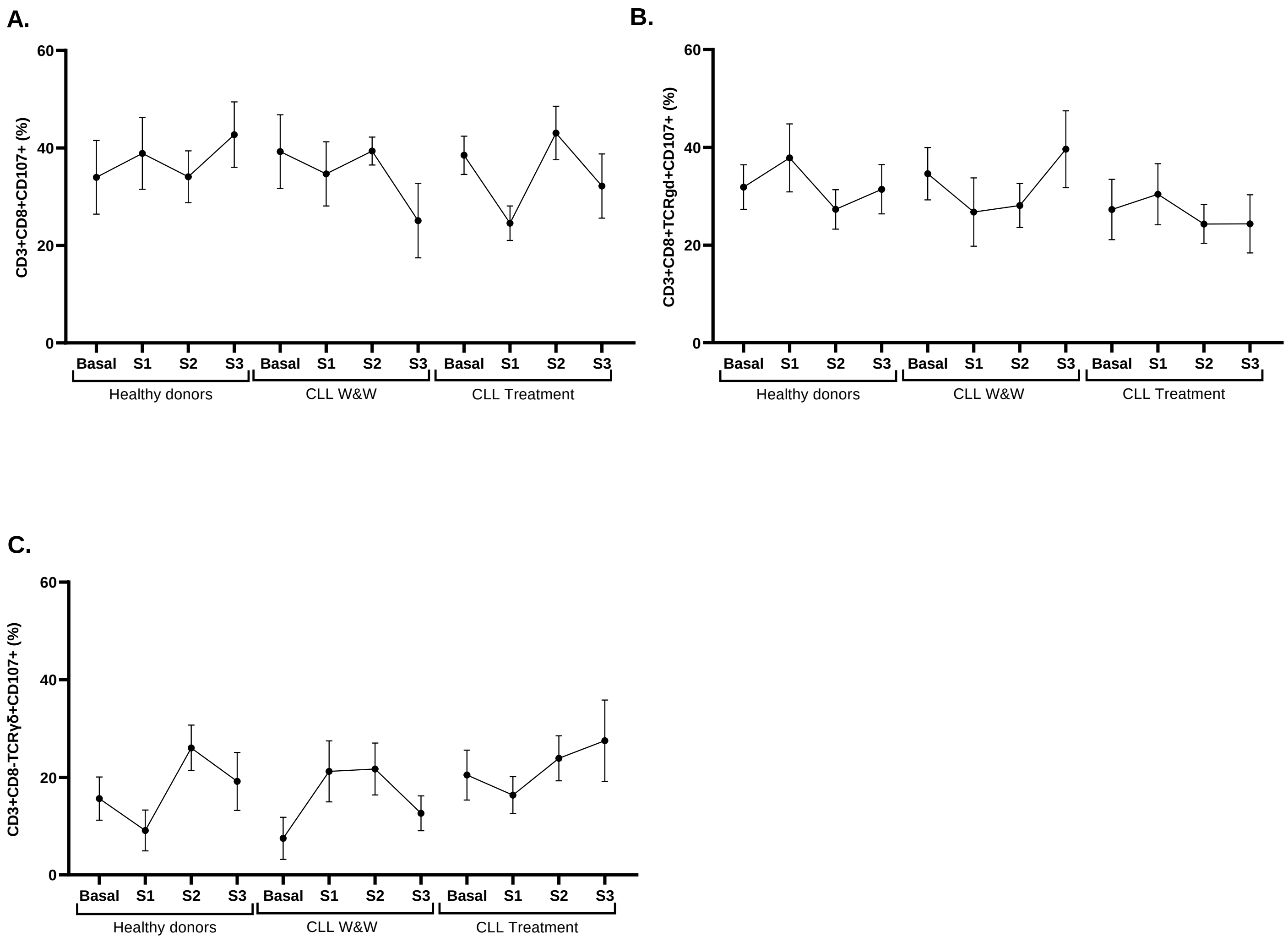

Supplement: Supplementary file 2 [file Image2.pdf]
