# Supplementary material for: Immune response dynamics of SARS-CoV-2 vaccination in chronic lymphocytic leukemia individuals: a descriptive analysis
Source: Front Immunol. 2025 Jun 6;16:1571680. doi: 10.3389/fimmu.2025.1571680 (PMC12179140; doi:10.3389/fimmu.2025.1571680)

Supplemental Figure 3. Expression levels of the degranulation marker CD107a in cytotoxic cell populations of PBMCs from healthy donors and chronic lymphocytic leukaemia patients on treatment and under the watch and wait (W&W) strategy. The expression of CD107a was analyzed by flow cytometry in the surface of NK cells (A) and NK-like cells (B) of PBMCs isolated from individuals of the three groups. Each dot in the graphs corresponds to mean ± SEM. Statistical significance was calculated using Wilcoxon signed-rank test, Mann-Whitney U and Student t test, as appropriate.

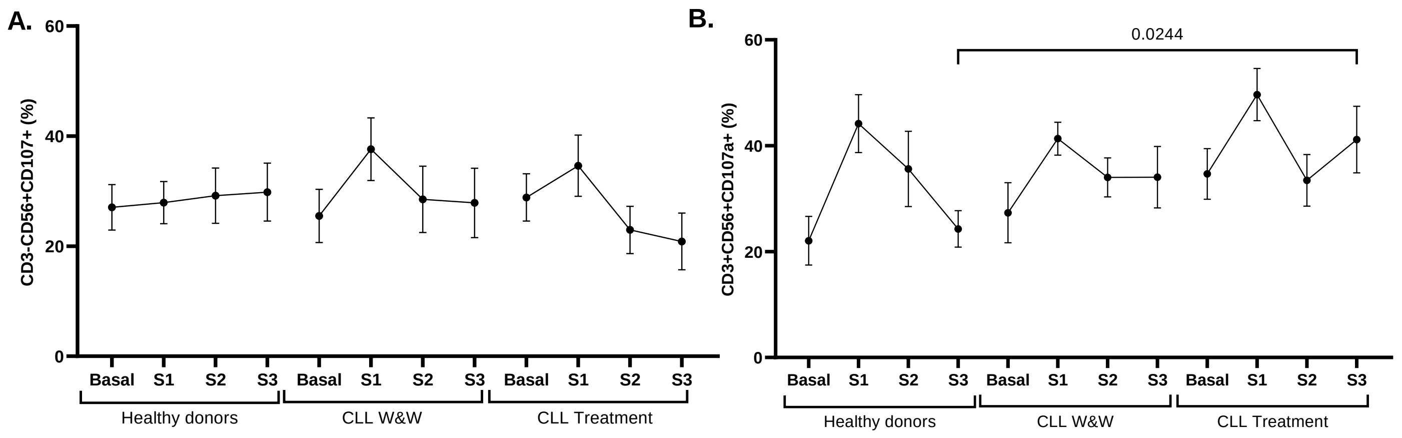

Supplement: Supplementary file 3 [file Image3.pdf]
